# Adaptive Immune Deficiency Impairs Neural Activity After Training and Retrieval

**DOI:** 10.3389/fnins.2021.739580

**Published:** 2021-11-15

**Authors:** Huiping Li, Zhongxiao Fu, Meixin Hu, Xiu Xu

**Affiliations:** ^1^Department of Child Health Care, Children’s Hospital of Fudan University, National Children’s Medical Center, Shanghai, China; ^2^Department of Neuroscience, University of Virginia, Charlottesville, VA, United States

**Keywords:** adaptive immune, c-Fos, neural activity, contextual fear conditioning, neurodevelopmental disorders

## Abstract

Neuroimmune interactions have been studied for decades. Several neurodevelopmental disorders have been associated with immune dysfunction. However, the effects of immune system on neuronal function remain unknown. Herein, based on c-Fos protein expression, we characterized the brain areas that are activated after contextual fear conditioning (CFC) training or retrieval in severe combined immune deficiency (SCID) and wild-type mice. Further, we analyzed the interregional correlations of c-Fos activity that are affected by deficiency in adaptive immunity. Results showed significantly lower c-Fos density in learning and memory-associated brain regions of SCID mice after memory retrieval, but not during the CFC training. Moreover, SCID mice exhibited remarkably discordant interregional neuronal activities of learning neuron circuits after CFC training, which could be the cause of inefficient activation of the memory circuit after retrieval. These results provide a new perspective on how adaptive immunity affects neuronal function. Adaptive immune deficiency impairs the coordination of neural activity after training and retrieval, which might be a potential therapeutic target for neurodevelopmental disorders.

## Introduction

A wide range of neurodevelopmental disorders, including autism spectrum disorder (Hughes et al., 2018) and neurodevelopmental delay (Bodnar et al., 2020), have been associated with immune system dysregulation. Advances in our understanding of neuroimmune interactions have led to fundamental changes in the concepts of immunology and neuroscience. The immune system is not self-regulated, but functions in close association with the nervous system (Dantzer, 2018). The nervous system regulates immune factors through neuroendocrine mediators and peripheral nerve endings innervating immune organs. Reciprocally, immune cells and mediators play a regulatory role in the nervous system and participate in the elimination and plasticity of synapses during development, as well as in synaptic plasticity during adulthood (Tanabe and Yamashita, 2018; Morimoto and Nakajima, 2019).

Studies have demonstrated that adaptive immunity is implicated in brain functions, including learning and memory retrieval (Brynskikh et al., 2008; Baruch and Schwartz, 2013; Radjavi et al., 2014). The first evidence came from experiments that examined the cognitive ability of wild-type (WT) and severe combined immune deficiency (SCID, deficient in both T cell and B cell responses) mice, by using the Morris water maze, a hippocampal-dependent visuospatial learning/memory task (Kipnis et al., 2004). SCID mice manifested significant impairment of spatial memory compared to their WT counterparts, and the brain function in SCID mice was restored when adaptive immunity was enhanced. Subsequently, researchers found that depletion of adaptive immune cells in naive mice also impairs spatial memory, supporting the notion that immunity plays a lifelong role in maintaining brain functions (Wolf et al., 2009; Derecki et al., 2010). However, the mechanism by which adaptive immunity affects memory is poorly understood.

Memory is a complex process through which information acquired during learning is stored. Synaptic and system changes are involved in the memory processes. The synaptic change is completed within hours of training and involves the stabilization of synaptic connectivity in localized circuits (Takeuchi et al., 2014). System changes are a more prolonged process that involves gradual reorganization of the brain regions that support memory (Frankland and Bontempi, 2005). Contextual fear conditioning (CFC) behavioral tests have been well studied as hippocampus-dependent spatial and associative learning tasks that activate hippocampal–cortical memory networks (Cowansage et al., 2014). Episodic memories initially require rapid synaptic plasticity within the hippocampus for their formation and are gradually consolidated in neocortical networks for permanent storage (Kitamura et al., 2017). Evidence shows that the hippocampus, amygdala, retrosplenial cortex (RSP), and prefrontal cortex are key regions supporting memory consolidation and retrieval processes (Tonegawa et al., 2018; de Sousa et al., 2019).

c-Fos is one of the first transcription factors whose induction was shown to be activity-dependent and is routinely used as an indicator of neuronal activation (Gallo et al., 2018; Silva et al., 2019). Fear memory paradigms have been shown to produce changes in c-Fos expression in different structures (Maviel et al., 2004; Lopez et al., 2012; Pignatelli et al., 2019). In this study, using c-Fos protein expression pattern, we characterized the brain areas that are activated in WT and SCID mice after CFC training or retrieval and evaluated the interregional correlations of c-Fos activity affected by adaptive immunity deficiency. Our results helped to uncover the immunity-neuron interaction and the potential therapy for neurodevelopmental disorders with immune dysfunction.

## Materials and Methods

### Mouse Strains and Housing

B6.CB17-*Prkdc^*scid*^/*SzJ (SCID, JAX 001913) and WT mice (C57BL/6J background) purchased from the Jackson Laboratory were maintained in the animal facility for at least 1 week prior to the start of the experiments. Mice were maintained and bred in-house under standard 12-h light–dark cycle conditions. They were given standard rodent chow and sterilized tap water *ad libitum*, unless stated otherwise. Male mice at 10–20 weeks of age were used for the behavioral experiments. Experimenters were blinded to the experimental groups during scoring and quantification. All experiments were approved by the Institutional Animal Care and Use Committee of the University of Virginia.

### Contextual Fear Conditioning Training and Retrieval

**FIGURE 1:**
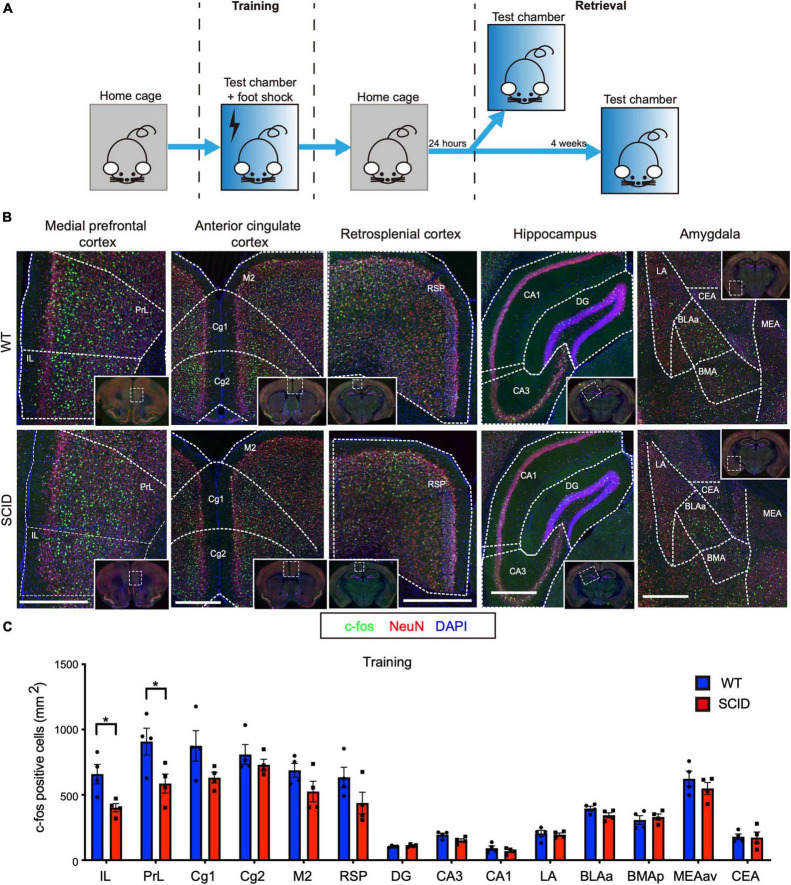
Limited differences in c-Fos expression between SCID and WT mice brain after contextual fear conditioning training. **(A)** General scheme of contextual fear conditioning training and retrieval. **(B)** Representative immunofluorescence images of the brain sections from WT (top) and SCID (bottom) mice 90 min after CFC training. c-Fos is in green, NeuN in red, and DAPI in blue. Brain regions: medial prefrontal cortex, anterior cingulate cortex, retrosplenial cortex, hippocampus, and amygdala. Scale bar, 500 μm. Low-power images were presented in the white solid line frames, with white dotted frames that showed the regions zoomed in. White dotted line outlined most of the brain regions for quantification. **(C)** c-Fos–positive cell density in different brain regions of WT and SCID mice (*n* = 4, data represent mean ± SEM, *p < 0.05).

The equipment used was purchased from Coulbourn Instruments (United States). Figure 1A illustrates the experimental design. During training, mice were introduced to a new context (test chamber), following which a stimulus was delivered in the form of a foot shock. The walls of the test chamber were painted white, and the chamber was scented with 0.25% benzaldehyde. Each mouse was placed in the test chamber for 3 min. After 3 min, electric foot shock (2 s, 0.50 mA) was delivered thrice during 198–200, 258–260, and 318–320 s. The mice were left in the test chamber for an additional 30 s before they were returned to their home cages.

At 1 day or 4 weeks posttraining, CFC-trained mice were returned to the test chamber for memory retrieval. The test at 1 day posttraining was referred to as recent retrieval. The test at 4 weeks posttraining was called remote retrieval. Mice were kept in the test cage for 3 min and returned back to their home cage without delivering the electric foot shock.

### Immunohistochemistry

Mice were euthanized by Euthasol overdose (10% vol/vol in saline, intraperitoneally) at 90 min (for c-Fos) and 60 min (for Arc) after training or retrieval. The animals were transcardially perfused with ice-cold phosphate-buffered saline (PBS) containing heparin (10 U mL^–1^) followed by 4% paraformaldehyde (PFA). Brains were dissected out and kept in 4% PFA overnight at 4°C. The fixed brains were washed with PBS and cryoprotected by immersing them in 30% sucrose solution for 48 h at 4°C. The fixed brain tissue was frozen in Tissue-Plus OCT compound (Thermo Scientific) and sliced into 40-μm-thick free-floating coronal sections using a cryostat (Leica). Brain sections were stored in PBS containing 0.02% sodium azide at 4°C until further use.

Brain sections were blocked with 1% bovine serum albumin (BSA), 2% normal serum (either goat or chicken), 0.2% Triton X-100, and 0.1% Tween 20 in PBS for 1 h at room temperature (RT). Sections were then incubated with appropriate primary antibodies in PBS containing 5% BSA and 0.2% Triton X-100 overnight at 4°C. The primary antibodies used were as follows: rabbit anti-c-Fos (1:1,000, Synaptic Systems, 226003), mouse anti-NeuN (1:300; Millipore, MAB377, clone A60), or rabbit anti-Arc (1:100, Santa Cruz, sc-15325). Brain sections were then washed three times for 15 min at RT with PBS containing 0.2% Triton X-100 and 0.1% Tween 20. This was followed by incubation at RT for 1 or 1.5 h with species-matched fluorescently conjugated secondary antibodies (1:1,000, Invitrogen) diluted in PBS containing 0.2% Triton X-100. Nuclei were stained with 4′,6-diamidino-2-phenylindole (DAPI, 1:10,000, Sigma-Aldrich) at RT for 15 min. The sections were washed with PBS three times for 15 min and mounted with Aqua-Mount (Thermo Scientific TA-125-AM) under coverslips.

### Image Acquisition and Analysis

For detecting c-Fos–positive cells, samples from the same experiment were stained and analyzed simultaneously using same settings on the Olympus FV1200 laser scanning confocal microscope with a 10× objective and 0.40 NA. Quantification of c-Fos expression was performed using the Fiji software (NIH). The following brain regions were analyzed using specific coordinates: infralimbic cortex (IL) and prelimbic cortex (PrL) at +1.98 to +1.54 mm from bregma; cingulate cortex (Cg1, Cg2, and M2) at +1.10 to +0.26 mm from bregma; hippocampus and RSP at −1.58 to −2.06 mm from bregma; and amygdala at −1.46 to −2.06 mm from bregma. Brain areas were manually outlined based on the DAPI and NeuN signals following the Allen Brain Reference Atlas and measured automatically by Fiji. The c-Fos–positive nuclei for each brain region were counted using the “Analyze Particles” tool in Fiji. Only the c-Fos–positive nuclei within a specific size range were counted, and a constant threshold level of staining was used to distinguish c-Fos–positive cells as follows: a fluorescence area between 18.07 and 117.78 μm^2^ and mean intensity greater than 60 (arbitrary units) were counted. This method has been described previously (Lopatina et al., 2014; Zhong et al., 2014). The density of c-Fos–positive cells (c-Fos per mm^2^) in each brain region was averaged from three sections per animal and at least four mice for each group.

### Statistical Analysis

Statistical analysis was performed using Prism 8.0 (GraphPad Software, Inc.). Comparison between two groups was performed using a two-tailed unpaired Student *t*-test. Pearson correlation coefficients were calculated for pairwise comparisons of c-Fos density among all 14 brain regions analyzed. Correlations were displayed as a color-coded correlation matrix generated using a custom R-code (R version 3.6.3).

## Results

### c-Fos Expression in Severe Combined Immune Deficiency and Wild-Type Mice After Contextual Fear Conditioning Training

Contextual fear conditioning behavioral test, a hippocampus-dependent spatial and associative learning/memory task, was used to compare the spatial learning/memory of SCID and WT mice. The CFC training was performed by transferring each mouse from the home cage to the test chamber where the animals received an electric foot shock (Figure 1A). SCID and WT mice were euthanized 90 min after CFC training. To investigate the brain regions that are activated during the training, brain slices were stained for c-Fos and NeuN. We quantified the density of c-Fos–positive cells in 14 brain regions that are known to play a crucial role in contextual fear learning and memory (Tovote et al., 2015). Figure 1B shows the density of c-Fos–positive cells in the IL, PrL, anterior cingulate cortex (Cg1, Cg2, and M2), RSP, hippocampus (DG, CA1, and CA3), and amygdala.

Although strong c-Fos expression was observed in these 14 brain regions of SCID mice, c-Fos expression was slightly higher in the WT mice brain. However, significant differences were observed only in the IL and PrL (*p* < 0.05, Figure 1C). IL and PrL regions have well-established roles in the fear memory circuit (Frankland and Bontempi, 2005).

### Inefficient c-Fos Expression in Severe Combined Immune Deficiency Mice Following Memory Retrieval

**FIGURE 2:**
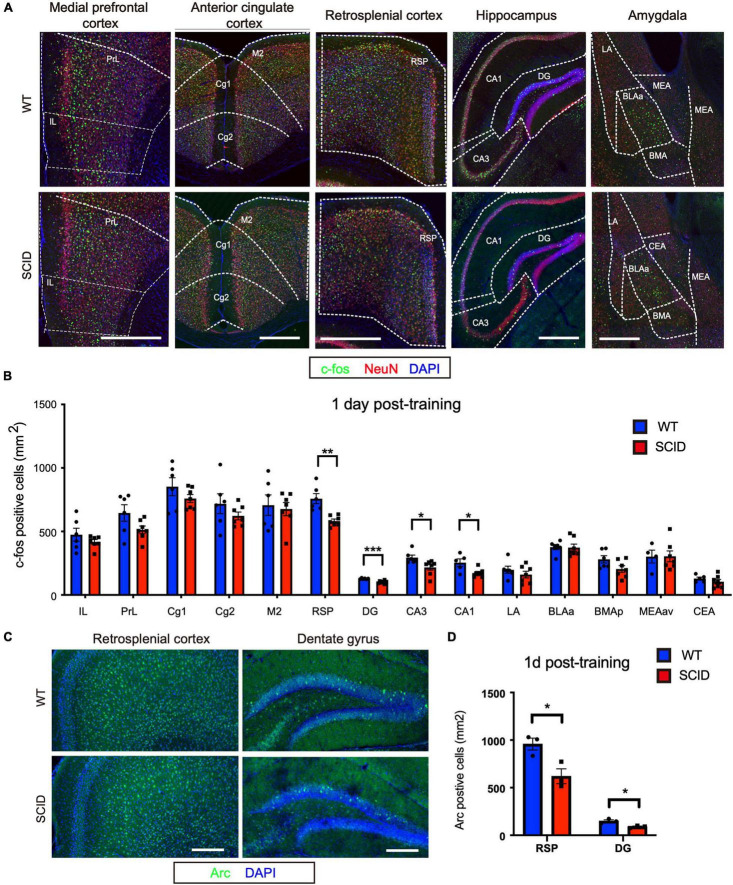
Inefficient neuronal activity in the hippocampus and retrosplenial cortex of SCID mice after recent memory retrieval. **(A)** Representative immunofluorescence images of five brain regions from WT and SCID mice after recent memory retrieval (1 day after CFC training). c-Fos is in green, NeuN in red, and DAPI in blue. Scale bar, 500 μm. **(B)** c-Fos–positive cell density in different brain regions of WT and SCID mice after recent memory retrieval (*n* = 6–7 per group, data represent mean ± SEM, *p < 0.05). **(C)** Representative immunofluorescence images of retrosplenial cortex and dentate gyrus from WT and SCID mice 60 min after recent memory retrieval. Arc is in green, and DAPI in blue. Scale bar, 200 μm. **(D)** Quantified Acr-positive cells at RSP and DG regions after recent memory retrieval (*n* = 3 per group; data represent mean ± SEM, *p < 0.05, **p < 0.01, ***p < 0.001).

To assess brain activity during recent memory retrieval, CFC-trained SCID and WT mice were transferred to the test cage at 1 day post-CFC training (Figure 1A). In the retrieval experiment, no electric shock was given to the animals. The animals were euthanized 90 min later, and c-Fos expression in the brain was evaluated (Figure 2A). Significant differences were observed in the hippocampus (DG, CA1, and CA3; *p* < 0.001, *p* < 0.05, and *p* < 0.05, respectively) and RSP (*p* < 0.01) (Figure 2B). Furthermore, the density of Acr (another immediate early gene)–positive cells at RSP and DG regions was lower in SCID mice than WT, which was consistent with c-Fos expression (*p* < 0.05) (Figures 2C,D).

**FIGURE 3:**
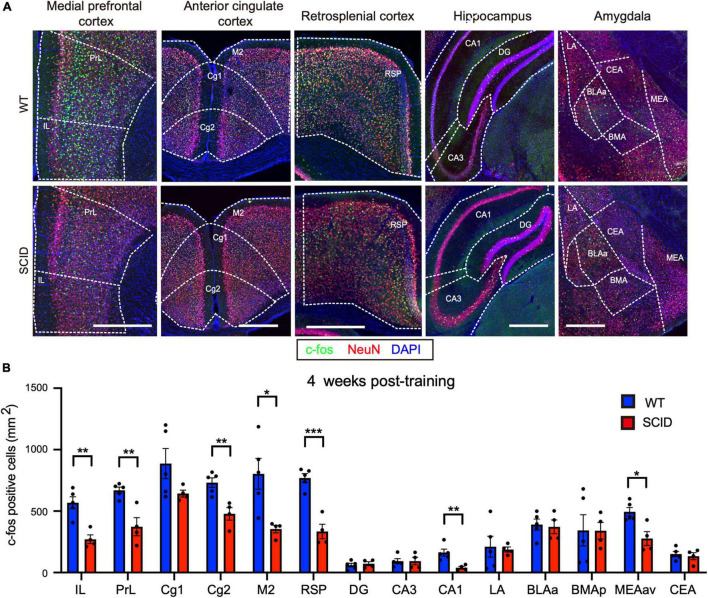
Significantly lower neuronal activity in memory-associated brain regions of SCID mice after remote memory retrieval. **(A)** Representative immunofluorescence images of five different brain regions from WT and SCID mice after remote memory retrieval (4 weeks after CFC training). c-Fos is in green, NeuN in red, and DAPI in blue. Scale bar, 500 μm. **(B)** c-Fos–positive cell density in the brain regions of WT and SCID mice after remote memory retrieval (*n* = 4–5 per group; data represent mean ± SEM, *p < 0.05, **p < 0.01, ***p < 0.001).

Remote memory retrieval was performed at 4 weeks post-CFC training (Figure 1A). CFC-trained SCID and WT mice were placed in the test cage without giving the electric shock. Results showed significantly lower c-Fos levels in SCID mice, especially in the medial PrL, RSP, and CA1 (Figures 3A,B).

### Lack of Adaptive Immunity Impairs the Interregional Activation of Learning and Memory Neuron Circuits

To gain insight into the functional connections within the learning and memory neuron circuits, we computed the correlation between each pair of brain regions in each group. The interregional correlation matrix for each experimental group led to the identification of sets of regions whose c-Fos density covaried across mice.

**FIGURE 4:**
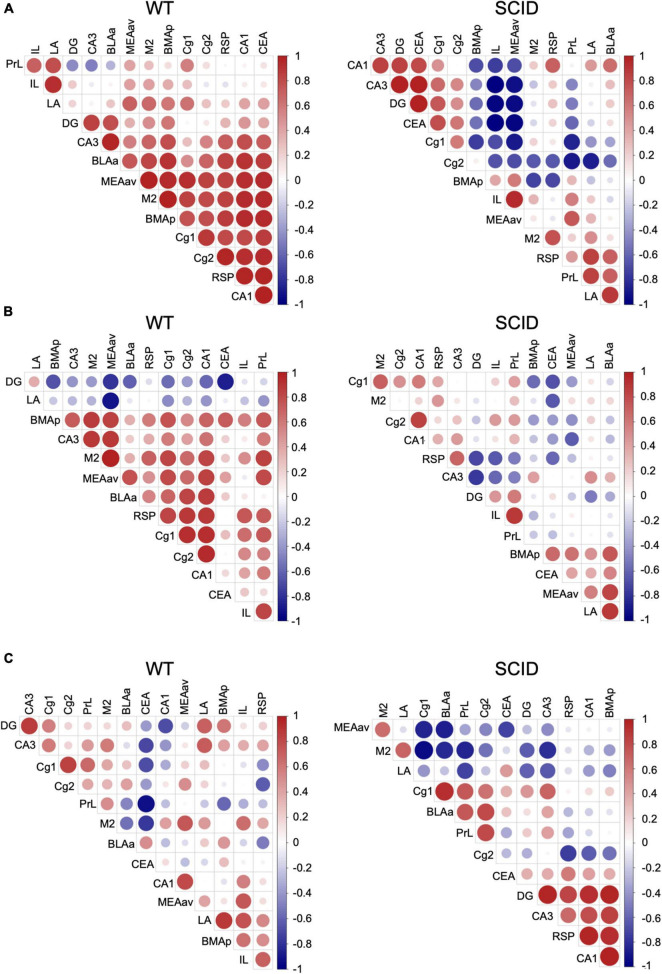
Variations in interregional correlation of c-Fos activation between WT and SCID mice. Pearson correlation matrices showing interregional correlations of c-Fos activation density. Axes represent the brain regions. The different colors represent Pearson correlation coefficients (scale, right). Panel **(A)** is for WT and SCID mice after the CFC training. Panel **(B)** represents WT and SCID mice after recent retrieval. Panel **(C)** represents WT and SCID mice after remote retrieval.

Results showed that in WT mice after CFC training, there was a strong connection between the brain regions that are important for learning and memory (Figure 4A, left). However, SCID mice showed remarkably discordant activation between the hippocampus–prefrontal cortex, hippocampus–amygdala, and prefrontal cortex–amygdala (Figure 4A, right). With the withdrawal of DG activation in memory retrieval, the prefrontal cortex to process memories might mirror that of the hippocampus to process recent memories. Inefficient activation of the cortex regions during the CFC training affected the memory retrieval, especially remote memory retrieval (Figures 4B,C).

## Discussion

To assess brain function and memory processes in mice lacking adaptive immunity, we compared c-Fos expression in 14 brain regions of SCID and WT mice that are known to be involved in the acquisition of contextual memories. IL and PrL showed remarkably lower c-Fos expression during learning in SCID mice. In recent memory retrieval test at 1 day post-CFC training, remarkable differences in c-Fos density in the RSP and hippocampus were observed between SCID and WT mice. In the remote memory retrieval test at 4 weeks post-CFC training, the prefrontal cortex, RSP, CA1, and medial nuclei of the amygdala of the SCID mice showed significantly lower c-Fos expression than WT mice.

Memories are thought to be initially stored within the hippocampal–amygdala network (recent memory) and, over time, slowly consolidate within the neocortex for permanent storage (remote memory) (Frankland and Bontempi, 2005; Lopez et al., 2012; Tonegawa et al., 2018; Todd et al., 2019). Consistent with previous studies, our study found that in WT mice, the DG-CA3 hippocampal network was active during learning and recent memory retrieval (1 day posttraining), whereas it was not activated by remote memory retrieval (4 weeks posttraining). Hippocampal CA1 activity was involved in both recent and remote memory retrieval. In our study, significantly lower neuronal activity was observed in the hippocampus and neocortex of the SCID mice during memory retrieval.

Furthermore, interregional activation patterns showed that SCID mice have abnormalities in neural circuits, which might be the cause of memory dysfunctions. The low neuronal activity in SCID mice was limited to IL and PrL regions during the learning stage. However, remarkable discordant activation was observed between the hippocampus–prefrontal cortex, hippocampus–amygdala, and prefrontal cortex–amygdala. IL and PrL, as part of the prefrontal cortex, are highly interconnected with the hippocampus, sensory cortex, motor cortex, and limbic cortex; therefore, they are ideally suited to integrate and synthesize information from a large number of different sources (Euston et al., 2012). Kitamura et al. (2017) found that neocortical prefrontal memory engram cells are rapidly generated during initial learning through inputs from both the hippocampus and the basolateral amygdala and became functionally mature with time, which is critical for remote contextual fear memory. The insufficient activation of IL and PrL by the hippocampus–prefrontal cortex circuitry does not affect the learning ability of SCID mice, but could have a potential effect on memory retrieval. The importance of interregional coactivation in neuron circuits has been demonstrated using chemogenetic neuronal inhibition and computational modeling (Kitamura et al., 2017; Roy et al., 2017; Pignatelli et al., 2019).

The findings of this study have to be seen in light of some limitations. In the CFC behavior test, SCID mice did not show significant difference for freezing time between genders. Considering stable behavior phenotype, we used only male mice in the study. However, immune deficiency and neurodevelopmental disorders affect both male and female. It is better to include female mice in a future study. The second limitation concerns that the study did not provide mechanistic insights into the impairments of neural activity in SCID mice after training and retrieval. Further studies will be aimed at understanding the molecular basis that underlies how adaptive immunity impairs neural activity.

Overall, our results provide new details about how adaptive immunity affects neuronal function. Mice lacking components of adaptive immunity have problems with neuronal activation and interregional coordination of neuron circuits during training and memory retrieval. Our results uncover the neural–immune interactions and will help in developing novel therapies for neurodevelopmental disorders due to immune dysfunction.

## Data Availability Statement

The raw data supporting the conclusions of this article will be made available by the authors, without undue reservation.

## Ethics Statement

The animal study was reviewed and approved by the Institutional Animal Care and Use Committee of the University of Virginia.

## Author Contributions

HL performed and analyzed IHC experiments and took the lead in writing the manuscript. ZF performed the behavioral tests. MH took part in c-Fos data analysis. XX conceived the project and provided advice. All authors provided feedback and contribution to the final manuscript.

## Conflict of Interest

The authors declare that the research was conducted in the absence of any commercial or financial relationships that could be construed as a potential conflict of interest.

## Publisher’s Note

All claims expressed in this article are solely those of the authors and do not necessarily represent those of their affiliated organizations, or those of the publisher, the editors and the reviewers. Any product that may be evaluated in this article, or claim that may be made by its manufacturer, is not guaranteed or endorsed by the publisher.

## References

[B1] Baruch K. Schwartz M. (2013). CNS-specific T cells shape brain function via the choroid plexus. *Brain Behav. Immun.* 34 11–16. 10.1016/j.bbi.2013.04.002 23597431

[B2] Bodnar T. S. Raineki C. Wertelecki W. Yevtushok L. Plotka L. Granovska I. (2020). Immune network dysregulation associated with child neurodevelopmental delay: modulatory role of prenatal alcohol exposure. *J. Neuroinflammation* 17:39. 10.1186/s12974-020-1717-8 31992316 PMC6988366

[B3] Brynskikh A. Warren T. Zhu J. Kipnis J. (2008). Adaptive immunity affects learning behavior in mice. *Brain Behav. Immun.* 22 861–869. 10.1016/j.bbi.2007.12.008 18249087

[B4] Cowansage K. K. Shuman T. Dillingham B. C. Chang A. Golshani P. Mayford M. (2014). Direct reactivation of a coherent neocortical memory of context. *Neuron* 84 432–441. 10.1016/j.neuron.2014.09.022 25308330 PMC4372249

[B5] Dantzer R. (2018). Neuroimmune interactions: from the brain to the immune system and vice versa. *Physiol. Rev.* 98 477–504. 10.1152/physrev.00039.2016 29351513 PMC5866360

[B6] de Sousa A. F. Cowansage K. K. Zutshi I. Cardozo L. M. Yoo E. J. Leutgeb S. (2019). Optogenetic reactivation of memory ensembles in the retrosplenial cortex induces systems consolidation. *Proc. Natl. Acad. Sci. U.S.A.* 116 8576–8581. 10.1073/pnas.1818432116 30877252 PMC6486739

[B7] Derecki N. C. Cardani A. N. Yang C. H. Quinnies K. M. Crihfield A. Lynch K. R. (2010). Regulation of learning and memory by meningeal immunity: a key role for IL-4. *J. Exp. Med.* 207 1067–1080. 10.1084/jem.20091419 20439540 PMC2867291

[B8] Euston D. R. Gruber A. J. McNaughton B. L. (2012). The role of medial prefrontal cortex in memory and decision making. *Neuron* 76 1057–1070. 10.1016/j.neuron.2012.12.002 23259943 PMC3562704

[B9] Frankland P. W. Bontempi B. (2005). The organization of recent and remote memories. *Nat. Rev. Neurosci.* 6 119–130. 10.1038/nrn1607 15685217

[B10] Gallo F. T. Katche C. Morici J. F. Medina J. H. Weisstaub N. V. (2018). Immediate early genes, memory and psychiatric disorders: focus on c-Fos, Egr1 and Arc. *Front. Behav. Neurosci.* 12:79. 10.3389/fnbeh.2018.00079 29755331 PMC5932360

[B11] Hughes H. K. Mills Ko E. Rose D. Ashwood P. (2018). Immune dysfunction and autoimmunity as pathological mechanisms in autism spectrum disorders. *Front. Cell Neurosci.* 12:405. 10.3389/fncel.2018.00405 30483058 PMC6242891

[B12] Kipnis J. Cohen H. Cardon M. Ziv Y. Schwartz M. (2004). T cell deficiency leads to cognitive dysfunction: implications for therapeutic vaccination for schizophrenia and other psychiatric conditions. *Proc. Natl. Acad. Sci. U.S.A.* 101 8180–8185. 10.1073/pnas.0402268101 15141078 PMC419577

[B13] Kitamura T. Ogawa S. K. Roy D. S. Okuyama T. Morrissey M. D. Smith L. M. (2017). Engrams and circuits crucial for systems consolidation of a memory. *Science* 356 73–78. 10.1126/science.aam6808 28386011 PMC5493329

[B14] Lopatina O. Yoshihara T. Nishimura T. Zhong J. Akther S. Fakhrul A. A. (2014). Anxiety- and depression-like behavior in mice lacking the CD157/BST1 gene, a risk factor for Parkinson’s disease. *Front. Behav. Neurosci.* 8:133. 10.3389/fnbeh.2014.00133 24795584 PMC4001052

[B15] Lopez J. Herbeaux K. Cosquer B. Engeln M. Muller C. Lazarus C. (2012). Context-dependent modulation of hippocampal and cortical recruitment during remote spatial memory retrieval. *Hippocampus* 22 827–841. 10.1002/hipo.20943 21542054

[B16] Maviel T. Durkin T. P. Menzaghi F. Bontempi B. (2004). Sites of neocortical reorganization critical for remote spatial memory. *Science* 305 96–99. 10.1126/science.1098180 15232109

[B17] Morimoto K. Nakajima K. (2019). Role of the immune system in the development of the central nervous system. *Front. Neurosci.* 13:916. 10.3389/fnins.2019.00916 31551681 PMC6735264

[B18] Pignatelli M. Ryan T. J. Roy D. S. Lovett C. Smith L. M. Muralidhar S. (2019). Engram cell excitability state determines the efficacy of memory retrieval. *Neuron* 101 274.e275–284.e275. 10.1016/j.neuron.2018.11.029 30551997

[B19] Radjavi A. Smirnov I. Kipnis J. (2014). Brain antigen-reactive CD4+ T cells are sufficient to support learning behavior in mice with limited T cell repertoire. *Brain Behav. Immun.* 35 58–63. 10.1016/j.bbi.2013.08.013 24012647 PMC3858511

[B20] Roy D. S. Kitamura T. Okuyama T. Ogawa S. K. Sun C. Obata Y. (2017). Distinct neural circuits for the formation and retrieval of episodic memories. *Cell* 170 1000.e1019–1012.e1019. 10.1016/j.cell.2017.07.013 28823555 PMC5586038

[B21] Silva B. A. Burns A. M. Graff J. (2019). A cFos activation map of remote fear memory attenuation. *Psychopharmacology* 236 369–381. 10.1007/s00213-018-5000-y 30116860 PMC6373197

[B22] Takeuchi T. Duszkiewicz A. J. Morris R. G. (2014). The synaptic plasticity and memory hypothesis: encoding, storage and persistence. *Philos. Trans. R. Soc. Lond. B Biol. Sci.* 369:20130288. 10.1098/rstb.2013.0288 24298167 PMC3843897

[B23] Tanabe S. Yamashita T. (2018). The role of immune cells in brain development and neurodevelopmental diseases. *Int. Immunol.* 30 437–444. 10.1093/intimm/dxy041 29917120

[B24] Todd T. P. Fournier D. I. Bucci D. J. (2019). Retrosplenial cortex and its role in cue-specific learning and memory. *Neurosci. Biobehav. Rev.* 107 713–728. 10.1016/j.neubiorev.2019.04.016 31055014 PMC6906080

[B25] Tonegawa S. Morrissey M. D. Kitamura T. (2018). The role of engram cells in the systems consolidation of memory. *Nat. Rev. Neurosci.* 19 485–498. 10.1038/s41583-018-0031-2 29970909

[B26] Tovote P. Fadok J. P. Luthi A. (2015). Neuronal circuits for fear and anxiety. *Nat. Rev. Neurosci.* 16 317–331. 10.1038/nrn3945 25991441

[B27] Wolf S. A. Steiner B. Akpinarli A. Kammertoens T. Nassenstein C. Braun A. (2009). CD4-positive T lymphocytes provide a neuroimmunological link in the control of adult hippocampal neurogenesis. *J. Immunol.* 182 3979–3984. 10.4049/jimmunol.0801218 19299695

[B28] Zhong J. Liang M. Akther S. Higashida C. Tsuji T. Higashida H. (2014). c-Fos expression in the paternal mouse brain induced by communicative interaction with maternal mates. *Mol. Brain* 7:66. 10.1186/s13041-014-0066-x 25208928 PMC4172782

